# Touchscreen typing pattern analysis for remote detection of the depressive tendency

**DOI:** 10.1038/s41598-019-50002-9

**Published:** 2019-09-16

**Authors:** Rafail-Evangelos Mastoras, Dimitrios Iakovakis, Stelios Hadjidimitriou, Vasileios Charisis, Seada Kassie, Taoufik Alsaadi, Ahsan Khandoker, Leontios J. Hadjileontiadis

**Affiliations:** 1 Department of Electrical and Computer Engineering, Aristotle University of Thessaloniki, Thessaloniki, Greece; 2 American Center for Psychiatry and Neurology, Abu Dhabi, UAE; 3 Healthcare Engineering Innovation Center, Department of Biomedical Engineering, Khalifa University of Science and Technology, Abu Dhabi, UAE; 4 Department of Electrical and Computer Engineering, Khalifa University of Science and Technology, Abu Dhabi, UAE

**Keywords:** Signs and symptoms, Biomedical engineering

## Abstract

Depressive disorder (DD) is a mental illness affecting more than 300 million people worldwide, whereas social stigma and subtle, variant symptoms impede diagnosis. Psychomotor retardation is a common component of DD with a negative impact on motor function, usually reflected on patients’ routine activities, including, nowadays, their interaction with mobile devices. Therefore, such interactions constitute an enticing source of information towards unsupervised screening for DD symptoms in daily life. In this vein, this paper proposes a machine learning-based method for discriminating between subjects with depressive tendency and healthy controls, as denoted by self-reported Patient Health Questionnaire-9 (PHQ-9) compound scores, based on typing patterns captured in-the-wild. The latter consisted of keystroke timing sequences and typing metadata, passively collected during natural typing on touchscreen smartphones by 11/14 subjects with/without depressive tendency. Statistical features were extracted and tested in univariate and multivariate classification pipelines to reach a decision on subjects’ status. The best-performing pipeline achieved an *AUC* = 0.89 (0.72–1.00; 95% Confidence Interval) and 0.82/0.86 sensitivity/specificity, with the outputted probabilities significantly correlating (>0.60) with the respective PHQ-9 scores. This work adds to the findings of previous research associating typing patterns with psycho-motor impairment and contributes to the development of an unobtrusive, high-frequency monitoring of depressive tendency in everyday living.

## Introduction

Mental illnesses are marked as the single largest burden of global disabilities, denoted by years lived with disabilities, whereas depressive disorder (DD) is considered the most common mental illness with an estimated share of 25–30%^1^. Moreover, DD is considered the leading factor^2^ for 10 million suicide attempts every year. Although active treatment for DD patients exists and significantly improves their quality of life, while reducing suicide risk^3,4^, the lack of resources and social stigma impedes^5^ diagnosis, limiting the number of treated cases. DD can manifest with various symptoms^6^, e.g., sadness, loss of energy and increased fatigue, causing patients’ psychomotor behaviour^7^ to differentiate from healthy population’s, in terms of gross motor activity, body movements and speech^8^. Clinical diagnosis involves diagnostic instruments, like Diagnostic Statistical Manual of Mental Disorders^9^, and standardized rating scales regarding symptoms that patients might face, with the most common ones being the Beck Depression Inventory^10^, the Hamilton Rating Scale for Depression^11^ the Major Depression Inventory^12^ and the Patient Health Questionnaire (PHQ-9)^13,14^. During this kind of assessment, many questions and statements elicit self-reports of subjective nature that may introduce bias to the ratings, whereas honesty and anonymity is suggested to improve the validity of the answers^15,16^. Raising awareness on DD and other mental illnesses and establishing new, objective methods for mental health monitoring could assist in eradicating barriers and motivating DD patients to reach out for professional care.

As mental health is becoming a major plague in modern societies, research efforts have been made to create digital methods for monitoring mental health and mood state. User interaction with modern computers and mobile devices can generate multi-modal and rich data, encapsulating patterns that can be associated with both motor and cognitive user states. In this direction, researchers have analysed user-interaction data, aiming at objectively monitoring mood and mental disorders. Data in focus included online social media activities^17^, text context^18^, audiovisual recordings^19,20^, GPS location^20,21^ and keystroke-related data^18,22–25^. Keystroke timing data, in particular, also referred to as keystroke dynamics, have been previously leveraged for the detection of psychomotor impairment, as the latter can affect typing cadence, which essentially consists of dense, coordinated finger movements. Kołakowska^26^ reviewed various studies, focusing on emotion recognition based on keystrokes dynamics, that showed promising results. Furthermore, Giancardo *et al*.^27^ examined keystroke dynamics variables for detecting transient psycho-motor impairment in healthy subjects due to sleep inertia, highlighting the discriminatory potential of statistical features from key hold time (HT) data. Additional studies^28,29^ extended the knowledge, by developing methods to detect motor skills decline caused by Parkinson’s disease based on smartphone typing activity; in their work, they also estimate motor symptoms severity, even when data are captured in-the-wild^30^. Moreover, Zulueta *et al*.^31^ reported statistical relationship between keystroke meta-data and mood disturbances in subjects with bipolar disorders. Finally, the recent approach of Cao *et al*.^32^, involving the fusion of keystroke timing information, accelerometer data and special characters typed, yielded promising results in terms of prediction of depression scores from bipolar patients and healthy controls, yet without providing insights on interpretability. Their approach was based on a typing session level and the use of deep learning, involving each subject’s data, both in the training and evaluation phases, while requiring at least 400 valid typing for converging to accurate results.

**Figure 1:**
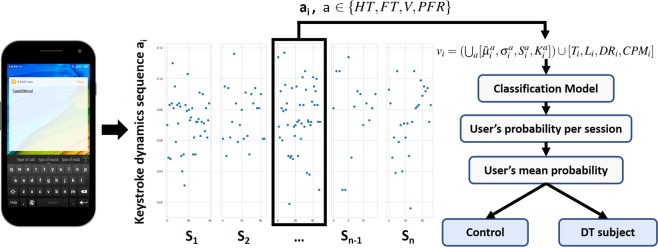
Feature vector extraction and classification pipeline. With respect to keystroke dynamics, each *i*_*th*_ subject’s typing session, i = *1, 2, …, n*, is represented by the hold time (HT), flight time (FT), speed (SP) and press-flight-rate (PFR) sequences *a*_*i*_, where *a* ∈ {*HT*, *FT*, *SP*, *PFR*}. For each sequence *a*_*i*_, statistical features, i.e., median ${\tilde{\mu }}_{i}^{a}$, standard deviation ${\sigma }_{i}^{a}$, skewness ${S}_{i}^{a}$, and kurtosis ${K}_{i}^{a}$, are extracted. The feature vector representing each typing session is finally formed by the union of the statistical features of all keystroke dynamics variables and the typing metadata of the session (duration (T), length (L), delete rate (DR) and characters per minute (CPM)), i.e., ${v}_{i}=(\mathop{\cup }\limits_{a}\,[{\tilde{\mu }}_{i}^{a},{\sigma }_{i}^{a},{S}_{i}^{a},{K}_{i}^{a}])\cup \,[{T}_{i},{L}_{i},D{R}_{i},CP{M}_{i}]$. For a single subject, feature vectors of all his/her typing sessions are fed into the classification pipeline, which yields probabilities for each typing session separately and averages these probabilities in order to classify the subject as with depressive tendency or healthy control.

Stemming from the aforementioned, the current work investigates the univariate and multivariate classification potential of keystroke dynamics variables and typing metadata and proposes a machine learning-based method for discriminating between young subjects with and without depressive tendency (DT), based on longitudinal data captured in-the-wild. The data collection study was remotely conducted via a custom application, namely TypeOfMood (https://play.google.com/store/apps/details?id=typeofmood.imeh=en), with a keyboard developed for the Android Operating System (OS) that participants installed on their own smartphone devices. TypeOfMood included a digitized version of the PHQ-9 questionnaire that each participant had to answer as the initial step. PHQ-9 scores were, afterwards, used to categorize participants into the two groups, i.e., subjects with DT and healthy controls (HC). For typing-related data logging, the participants used the custom keyboard of TypeOfMood, which replaced the default typing input method across all applications and aspects of the OS. The custom keyboard recorded keystroke timing information, i.e., sequences of timestamps of key presses and releases, as well as the relative-to-screen pixel coordinates of each key pressed and typing metadata (delete rate, number of characters typed and typing session duration), in the background, without interfering with participants’ routine typing. Timestamp sequences collected were used to extract traditional keystroke dynamics variables, widely used in similar works that investigated relationships with psychomotor impairment, i.e., the hold time (HT - time interval between pressing and releasing a key) and flight time (FT - time interval between releasing a key and pressing the next one). In this work, we further introduced two novel variables, i.e., speed (SP - the distance between successive keys divided by the flight time) and press-flight-rate (PFR - the ratio between the HT of a key and the FT to the next one). Statistical features of keystroke dynamics variables, along with typing metadata, extracted on a typing session level, were used to evaluate a classification pipeline under a leave-one-subject-out (LOSO) cross-validation scheme. The latter yields the probability of a subject having DT or being HC, by averaging probabilities deduced from her/his single typing sessions. An illustration of the feature vector extraction and classification process is depicted in Fig. 1.

## Results

Regarding the dataset collected, the vast majority (76%) of typing-related data were captured while users typed messages on the Facebook Messenger application, while the rest were captured from typing on the Chrome mobile browser (5%), Instagram social media application (5%), WhatsApp messaging application (3%), and other applications (11%). Using the two-sided Mann-Whitney U test, no statistically significant difference was found between the two subject groups’ (with/without DT) distributions, as far as the percentage of application usage is concerned. In total, 34,581 typing sessions, corresponding to 234 hours of typing, were collected, with an average (std) of 55.14(50.08)/66.46(42.54) sessions per day, for subjects with/without DT, respectively.

**Figure 2:**
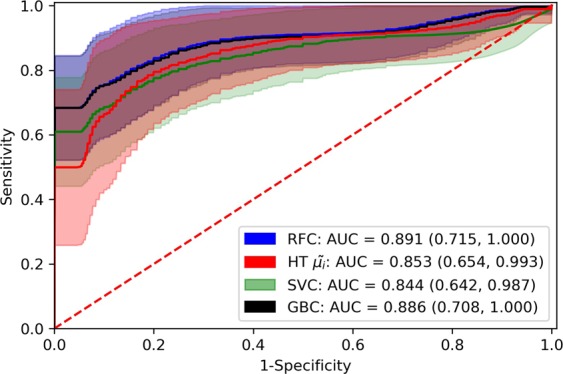
Comparison of Receiver Operating Characteristics (ROC) curves for different settings of the classification pipeline. ROC curves corresponding to Random Forest Classifier (RFC), Gradient Boosting Classifier (GBC) and Support Vector Machine Classifier (SVC), as well as of the best-performing feature in the univariate setting, i.e., ${{\tilde{\mu }}_{i}}^{HT}$, are illustrated. The solid lines represent the mean ROC curves, while the shaded areas represent the 95% Confidence Intervals (*CI*), computed over 1,000 bootstraps. The RFC-based classification pipeline (blue line and shade) achieved the highest Area Under the ROC Curve (*AUC* = 0.891) with the narrower *CI* (0.715–1.00).

**Figure 3:**
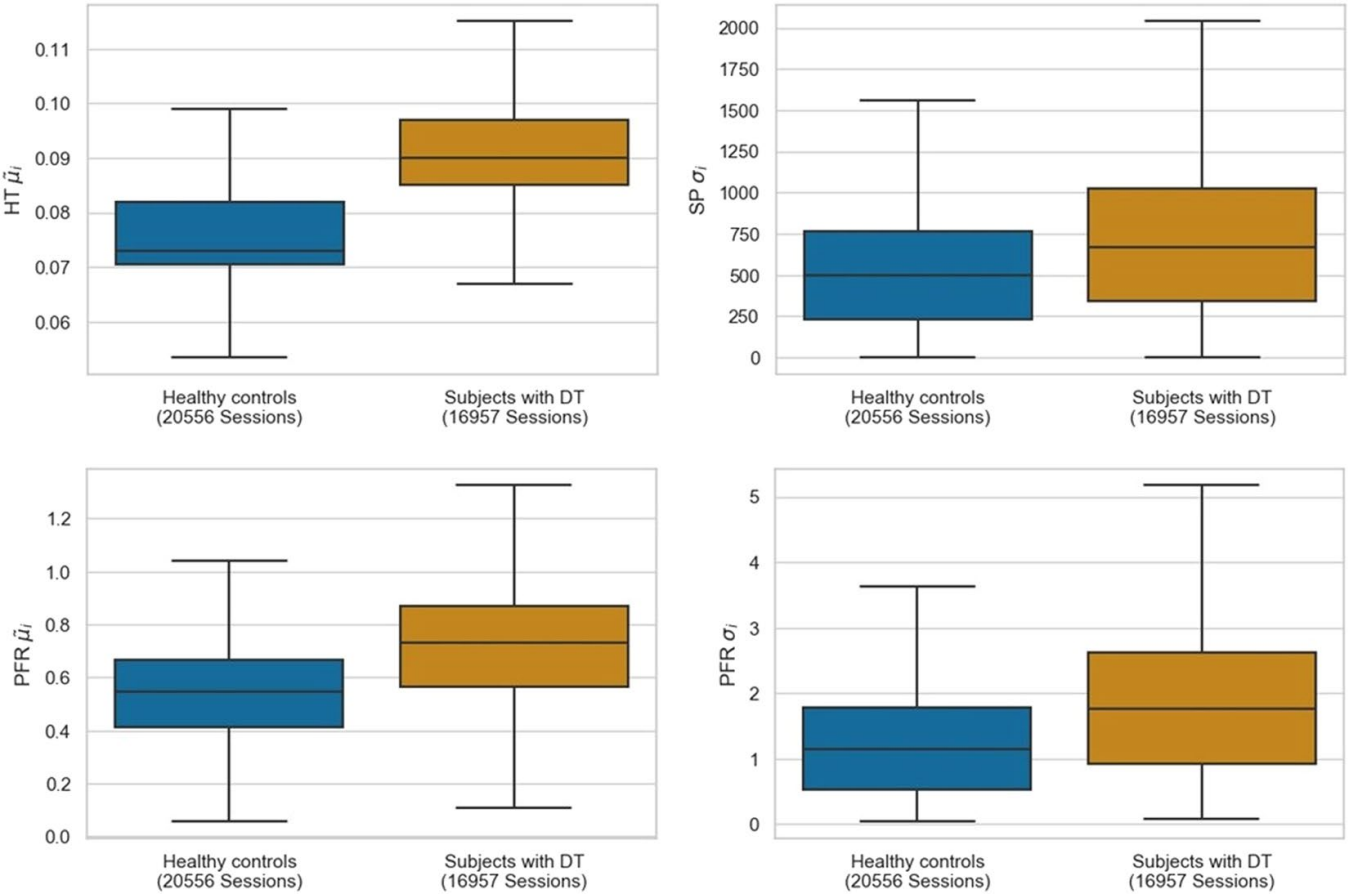
Group-wise comparison of distributions of the most consistently selected features. Box plots of HT ${\tilde{\mu }}_{i}$, SP *σ*_*i*_, PFR ${\tilde{\mu }}_{i}$ and PFR *σ*_*i*_ (see Table 1) are computed over 16,957/20,556 typing sessions of subjects with/without DT. Each box plot represents the median value (horizontal line within the box), the interquartile range (height of the box) computed from first (bottom) to third quartile (top), and the minimum/maximum value (end of “whisker” below/above box) within the interquartile range.

The performance of the proposed method was evaluated for different settings of the classification pipeline, which included three types of classifiers (Support Vector Machine Classifier^33^, Random Forest Classifier^34^ and Gradient Boosting Classifier^35^), feature selection and hyperparameter optimization, under the LOSO scheme, using Receiver Operating Characteristic (ROC) analysis. The best-performing pipeline, consisting of a Random Forest classifier and five-best feature selection, resulted in mean *AUC* = 0.89 [0.72–1.00; 95% Confidence Interval (CI)] with 0.82/86 sensitivity/specificity. ROC-based performance comparison of the different multivariate classification pipelines and the best univariate model, i.e., ${{\tilde{\mu }}_{i}}^{HT}$, achieving an *AUC* = 0.85 (0.65–0.99; CI) with 0.82/0.86 sensitivity/specificity, is depicted in Fig. 2. Furthermore, Table 1 presents the results of group-level (subjects with DT and HC) statistical comparisons (using a two-sided Mann-Whitney U test) for each feature, as well as its importance and frequency of selection over all LOSO iterations for the best-performing pipeline. It is observed that certain features of HT, SP and PFR are consistently selected (satisfying the selection criterion of ≥90%), thereby their discriminatory potential is highlighted. Figure 3 depicts a group-wise comparison of the distributions of the consistently selected features, where it is seen that subjects categorized as with DT exhibit higher average HTs and PFRs, as well as more variant SPs and PFRs, than HC, denoting slower movements of the former subject group in comparison with the latter.

**Table 1:** Results of group-wise statistical comparison, importance and frequency of selection for each feature and the best-performing classification pipeline.

Gray Feature		Statistical Significance	RFC Feature Importance (std)	Times selected %
HT	${\tilde{\mu }}_{i}$	p < 0.001	0.55 (0.02)	**100%**
	*σ*_*i*_	p < 0.001	0.08 (0.05)	72%
	*S*_*i*_	p < 0.001	0.01 (0.02)	4%
	*K*_*i*_	p < 0.001	—	0%
FT	${\tilde{\mu }}_{i}$	p < 0.001	0.01 (0.03)	4%
	*σ*_*i*_	p < 0.001	—	0%
	*S*_*i*_	p < 0.001	—	0%
	*K*_*i*_	p < 0.001	—	0%
SP	${\tilde{\mu }}_{i}$	p < 0.001	0.02 (0.01)	20%
	*σ*_*i*_	p < 0.001	0.11 (0.01)	**100%**
	*S*_*i*_	p = 0.001	—	0%
	*K*_*i*_	p < 0.001	—	0%
PFR	${\tilde{\mu }}_{i}$	p < 0.001	0.13 (0.01)	**100%**
	*σ*_*i*_	p < 0.001	0.10 (0.01)	**100%**
	*S*_*i*_	p = 0.083	—	0%
	*K*_*i*_	p < 0.001	—	0%
T		p < 0.001	—	0%
L		p < 0.001	—	0%
DR		p < 0.001	—	0%
CPM		p < 0.001	—	0%

Statistical differences between the two groups were evaluated using the non-parametric two-sided Mann-Whitney U test, applied on the averaged values of features per subject. Average (std) feature importance and frequency of selection (percentage of times selected) were derived from the Random Forest Classifier (RFC) using the GINI criterion and the *k*-best selection method, respectively, over all iterations of the leave-one-subject-out (25 subjects) validation process. The bold values indicate the features that pass the selection threshold, i.e., times selected ≥90%.

After conducting a logistic regression test with the prediction probability (outputted by the best-performing pipeline), age, education level, probability convergence, average number of typing sessions per day and gender as independent variables and the subject group (with DT and HC) as the dependent binary variable (Table 2), it was derived that the prediction probability is significantly associated with the subjects’ status (*p* < 0.05). On the contrary, the rest of the factors did not yield statistically significant effects. Finally, correlation analysis (Spearman’s correlation) was employed in order to investigate whether the outputted probabilities of the best-performing pipeline and the best-performing feature can scale along with PHQ-9 scores. Both variables were found to be significantly (*p* < 0.05) correlated with PHQ-9 scores, with the outputted probabilities of the best-performing pipeline yielding a correlation coefficient of 0.64, while the best-performing feature, i.e., ${{\tilde{\mu }}_{i}}^{HT}$, produced a correlation of 0.57, indicating, in both cases, a plausible analogous relationship with the PHQ-9 scale.

**Table 2:** Results of the Logistic Regression test.

Gray	Coefficient	Standard Error	z-value	Significance (*p*-value)
Constant	−6.9967	7.115	−0.983	n.s. (*p* = 0.325)
Age	0.0009	0.288	0.003	n.s. (*p* = 0.998)
Education	−3.5295	3.508	−1.006	n.s. (*p* = 0.314)
Gender	0.7664	1.562	0.491	n.s. (*p* = 0.624)
Convergence	0.0018	0.003	0.537	n.s. (*p* = 0.591)
Sessions per day	0.0342	0.023	1.488	n.s. (*p* = 0.137)
Prediction	11.8794	5.236	2.269	**sig**. (*p* < 0.05)

The prediction probability (outputted by the best-performing pipeline), age, education level, number of typing sessions required for probability convergence, average number of typing sessions per day and gender were used as independent variables and the subject group (with/without depressive tendency) as the dependent binary variable. Only prediction probabilities exhibit a statistically significant (*p* < 0.05) association with subjects’ status. n.s.: not significant; sig.: significant.

## Discussion

Although the health care sector has been transformed due to technological advances over the last decades, psychiatric care tends to adapt in a much slower pace due to the complexity of brain function that drives cognition, rendering diagnosis of mental disorders and monitoring a challenging task. In addition, economic factors and social stigma associated with mental illnesses discourage people from seeking professional help, resulting in numerous undiagnosed cases^36,37^; hence, such disorders, including DD, may remain untreated. Nevertheless, advances in the clinical decision-making process, through remote and objective monitoring of depressive symptoms based on information and communication technology (ICT) tools, set a promising research direction. The emergence of objective tools that aim to assist patients in self-managing their mental health could expunge the subjective factors that underlie the current practice of diagnosis, while enriching the information that the patient can share with the clinician. In particular, studies have reported patients’ willingness to use smartphone-based applications^38^ to monitor their mental health and be further consulted^39^ by health care professionals regarding diagnosis and management. Despite users’ positive incline towards such self-management tools, dropout rates of applications that require active user engagement are often high^40^, highlighting, therefore, the need of a design and functionality logic that facilitates long-term adherence. Passive recording of data may constitute a solution towards this direction and, at the same time, provide a rich source of behavioural information that may reveal patterns associated with the user’s mental health status, especially through analysis of complex interaction data with consumer devices, such as smartphones. The method proposed in this work was developed with the aim of overcoming the obstacle of frequent supervision or guidance to users and, at the same time, with respect to anonymity and privacy within an ecologically valid data capturing setting. Our key motivation is to contribute towards objective monitoring of mental disorders in everyday life, by advancing ICT-based screening tools that could facilitate diagnosis. The amount of data collected during our study, the fact that they were collected in-the-wild, and the classification performance achieved highlight the promising screening potential of our method, which collaterally fosters long-term adherence and scalability. Therefore, a tool incorporating our method could be used as an unobtrusive and high-frequency monitoring test of the patient’s status, in order to support health care professionals and encourage self-management of mental health in a discreet manner.

Based on the evaluation approach adopted here, training of classifiers in each LOSO iteration was based on a balanced training set, formed by feature vectors extracted on typing session level, which renders the method resilient to class imbalance, whereas it reduces further the risk of over-fitting due to the training in a large observation space. Three out of the four subjects that were misclassified by the method reported PHQ-9 scores near the threshold of 5 (two healthy controls with a PHQ-9 score of 3 and a subject with DT with a score of 5), which can be, partly, attributed to the error that the subjective nature of PHQ-9 questions induce. Moreover, correlation results between single features and prediction probabilities with the PHQ-9 scores highlight the potential of exploiting these data in combination with a regression model to quantify the severity of depressive symptoms, providing, in this way, a granular and objective estimation to the expert that monitors the DD patient’s status.

Classification of subjects was based on a threshold of 5 for the self-reported PHQ-9 score, in order to investigate the classification performance with mild depressive symptoms as the cutoff level. Nevertheless, since the optimal PHQ-9 cutoff point for major depression is 10^41,42^, the proposed method was also tested for this case. Selecting a PHQ-9 cutoff of 10 resulted in a re-categorization of subjects in the two groups of interest, i.e. 17 subjects with DT and eight HC. In this case, the best ROC-based performance of 0.81 (0.58–0.99; 95% CI) with sensitivity/specificity 0.75/0.82 is achieved via the Gradient Boosting Classifier. Although groups are more imbalanced, the latter results denote that the method can perform with similar results when identifying subjects that reported moderate depressive disorder.

**Figure 4:**
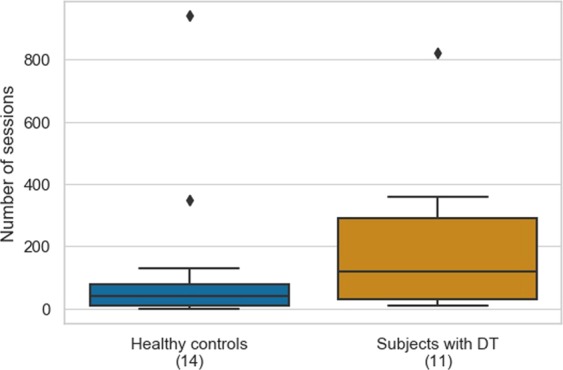
Group-wise comparison of the number of typing sessions required for prediction probability convergence. Convergence is considered here to be achieved when the cumulative distribution function of the prediction probabilities is lower than 0.05 of the subject’s average prediction probability, as outputted by the best-performing classification pipeline. There is no statistically significant difference between the two subjects groups, in terms of the number of typing sessions required for convergence, as derived by the Mann-Whitney two-sided test (*p* = 0.15).

**Figure 5:**
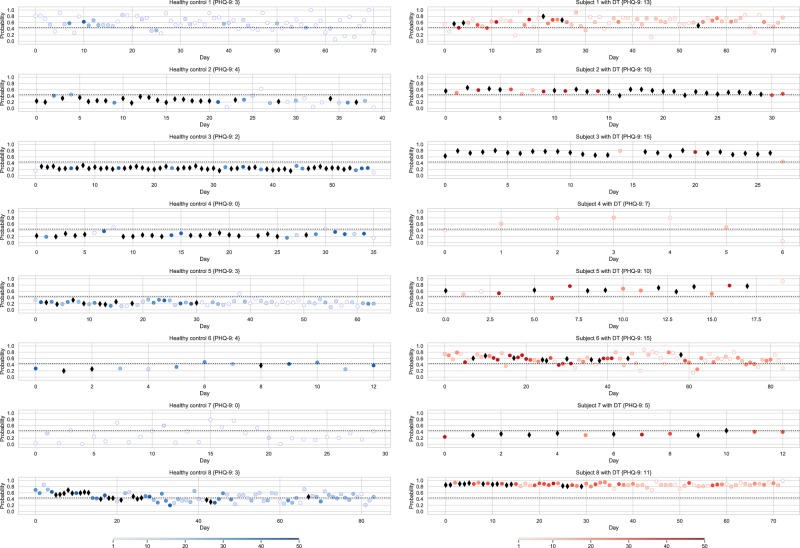
Indicative examples of the day-to-day variation of the estimated probability for subjects with depressive tendency and HC. Each sub-plot corresponds to the whole course of a subject’s data contribution, where the *y*-coordinate of the markers is the subject’s daily mean prediction probability outputted by the best-performing RFC-based classification pipeline and features representing typing sessions of the day (*x*-coordinate) as input. Blue and red colors denote HC and subjects with DT, respectively. Color opacity of each circle denotes the number of typing sessions of the subject during the day that ranges within [1–50], whereas the black diamond marker (◆) denotes a number of typing sessions >50; in both cases, these typing sessions were used in the classification pipeline to output the daily probability. The dashed horizontal line represents the Youden index-based^58^ classification threshold for equal misclassification cost, as computed from the overall prediction probabilities of all subjects.

The robustness of the proposed method is further reflected in the minimum number of typing sessions required for convergence to an accurate prediction probability. Unlike the minimum of 400 typing sessions with at least 10 keystrokes required in the approach of Cao *et al*.^32^, the proposed method can provide a stable estimation when 50 typing sessions with a minimum of eight keystrokes per session become available. In fact, as the number of typing sessions increases, the cumulative probability per subject does not differ more than 0.05 from the final estimation. A comparison of the number of sessions required for convergence per study group is shown in Fig. 4 and is class-independent, as statistically tested with the Mann-Whitney two-sided test. Additionally, Fig. 5 presents day-to-day variations of the prediction probability for indicative subjects, with respect, also, to the daily number of typing sessions. As it is seen from Fig. 5, in most cases, there is a notable fluctuation in the predictions. In order to investigate what causes this fluctuation based on the available data, the correlation between the standard deviation of the daily prediction probabilities with the average number of daily typing sessions was computed, resulting in a statistically significant (*p* < 0.05), negative correlation of −0.60. Based on the latter, the variability in prediction probabilities may be caused to a certain extent by the lack of enough data due to daily contributions of less than 50 typing sessions, which was shown to be required for convergence (e.g., healthy controls 1 and 7; subjects 1 and 4 with DT). On the contrary, subjects typing more within the day (>50 sessions-denoted with black diamonds in Fig. 5), e.g., healthy controls 3 and 4; subjects 2 and 8 with DT, yield less variant prediction probabilities. However, as Fig. 5 shows, despite the within-subject variation in daily typing sessions, the proposed approach is robust, in terms of categorizing subjects as HC or with DT, almost in all cases and days; hence, day-to-day variation in prediction probabilities does not impose a negative effect on classification performance. Nevertheless, monitoring of variation in the number of sessions per day could be useful to infer for the interaction of the user with the smartphone keyboard and, perhaps, contribute to the identification of any interaction pattern or alteration of any existing one. Such examples are depicted in Fig. 5, where HC 4 and subject 8 with DT exhibit a de crease in typing interaction after day 30. Of course, such observation needs further investigation in order to be associated with potential mood alteration or other external factors. Overall, longitudinal results presented in Fig. 5 reveal the potential, especially if combined with other data sources, of developing a system for monitoring psychomotor behavior and alerting, in case of significant changes towards DD, and/or as a monitoring tool of the effect of treatment on diagnosed patient’s symptoms.

Out of all features fed to the classification model, a key HT-based feature had the best univariate classification performance, i.e., ${{\tilde{\mu }}_{i}}^{HT}$ achieved *AUC* = 0.85 with 0.82/0.86 sensitivity/specificity, while the correlation between ${{\tilde{\mu }}_{i}}^{HT}$ and PHQ-9 scores was 0.57. These findings align with previous research regarding typing patterns and psychomotor impairment^27,30,43^ and are further explained by the kinematic and cognitive functions involved in typing and in particular, the action of key pressing^44–47^. As it is depicted in Fig. 3, subjects with DT exhibit longer HTs and have greater standard deviation of SP, when compared to HC. Specifically, subjects with DT had longer key HTs, indicating a slower motor reaction time, which can be linked to psychomotor retardation^48^ that is present in mental disorders. Additionally, slower reaction time of subjects with DT to audiovisual stimulus^49^ may have affected the HT variable that is influenced by the visual feedback of characters being registered in the case of touchscreen keyboards. On the other hand, the larger standard deviation of SP and PFR can be plausibly associated with mood swings that DD patients may experience^50^, and have been previously reported to affect DD patients in longitudinal studies^51,52^. Fusion of typing-related data with additional information, such as special-character keys and accelerometer data reflecting fine-motor skills, has been explored in similar studies^32^, with the developed methods exhibiting similar performance reported in this work; however, they lack interpretability and are data hungry, in terms of training and convergence.

Mental disorders affect non-motor and motor functional aspects of patients, whereas each mental disorder presents with different symptoms, revealing, in this way, the need for a multi-modal approach to a remote mental disorder monitoring tool. Based on the promising results presented here and the authors’ previous experience with multi-modal behavioral modelling in the context of the i-PROGNOSIS project (www.i-prognosis.eu), it is suggested that a future study investigating multi-modal unobtrusive sensing and digital markers for mental disorders should incorporate the requirement of long-term adherence, considering also the level of engagement of other remote large-scale studies^40^. In this vein, additional data sources that can be sensed in an unobtrusive manner have been reported in the relevant literature and linked with DD. A holistic approach can extend the current method by enriching the sensed data with physical^53^ and sleep activity information^54^, heart rate variability and diurnal activity^55^, as well as other informative data. The latter could be further exploited for clustering different cases of behavioural patterns that may be caused by different mental disorders.

Despite the promising results of the current method towards remote, unsupervised detection of DT in daily life, certain limitations exist. In particular, the number of subjects involved in the study, along with the absence of detailed medical history, limit the generalization of the results, in order to validate the diagnostic properties of the method. Nevertheless, the large number of typing sessions captured per subject compensates, in part, the aforementioned study size limitation. In addition, as the focus was placed on the cut-off of mild depressive symptoms, the self-reported PHQ-9 scores acquired ranged between 0 and 4 (from absence to minimal DT) for HC and from 5 to 15 (mild to moderately severe DT) for subjects with DT and, therefore, they did not cover the whole PHQ-9 spectrum [0–27]. From a holistic perspective, future inclusion of more subjects in the study, forming a uniformly distributed population in terms of the PHQ-9 scale, would contribute to the validation of the diagnostic properties and of the way the method scales up with depression severity, in a more precise manner. Finally, ordinary evaluation, in terms of the PHQ-9 test, takes place every two weeks, while during our study, the classification was based on subject categories that were formed based on PHQ-9 scores obtained at the first launch of the TypeOfMood application, followed by the period of typing-related data collection that lasted up to two months. As a future extension, periodically repeated PHQ-9 evaluations could be combined with results obtained across days (similar to those presented in Fig. 5), with the potential of providing a clearer indication of the subject’s status, in a dynamic way. This would allow for better monitoring of DT fluctuations and significant changes with time and for evaluating the performance of various interventions (e.g., medication, psychological support).

Methods, which employ unobtrusive information capturing towards unsupervised remote screening, should be aware-by-design of privacy, security and anonymity and comply with the relevant regulatory frameworks, as well as ethical guidelines on research. The proposed approach complies with the latter and yields promising results towards remote depressive tendency detection in young adults, based on typing patterns captured in-the-wild. Considering future adoption and extension, the diagnostic properties of the method proposed here are reported along with confidence intervals. The true value of each diagnostic performance probably lies within the span of the reported confidence interval, an assumption that can be validated as more participants join the study, which is facilitated by the scalable nature of the data collection process. Analysis of the statistical power needed to compare the two groups’ prediction means by a two-sided equality resulted in a power of 0.78 (significance level of 0.05) for the current sample size. A future study, aiming to confirm clinical validity of the developed method in terms of screening for DD, considering a prevalence of the disorder around 5%, will require a minimum sample size of 2,200 subjects (including 110 subjects with DT), in order to achieve a minimum power of 80% for detecting a change in sensitivity and specificity from 0.80 to 0.90 of a screening test, based on a target significance level of 0.05^56^.

Inferring psychomotor impairment from typing patterns probably cannot be used as a standalone method for differential diagnosis of DD, without the inclusion of any other source of information. However, it could yield objective evidence for health care experts, provided that a larger cohort is involved in the study. The inclusion of relevant tests in clinical practice could assist experts by serving as a complimentary tool for unobtrusive and remote monitoring of symptoms related with DD, facilitating decision making and encouraging individuals to self-manage their condition. In the future, the proposed approach could be further improved based on longitudinal data by a larger pool of subjects, who will frequently report their mood and physical state, with concurrent monitoring by medical experts, in order to investigate transitions towards depression or detect mood swings and drug-related fluctuations. The vision is to empower patients to self-manage their symptoms and provide mental health experts with objective information via frequent symptoms monitoring, which could lead to adaptive and more effective treatment. The latter is more feasible with remote studies involving unobtrusive data collection, as corroborated by the authors’ prior experience with a similar smartphone-based study on Parkinson’s Disease (i-PROGNOSIS)^30^. Due to the passive nature of data collection, a larger volume of data is collected and at the same time, long-term user adherence is achieved.

## Methods

**Figure 6:**
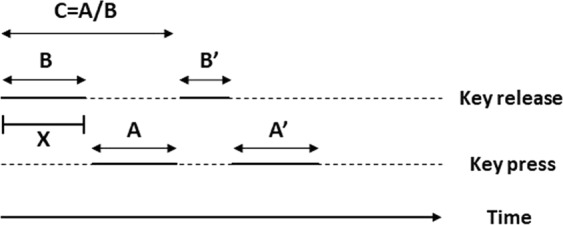
Representative illustration of the keystroke dynamics variables exploited in this work. A: Hold Time (HT), time interval between pressing and releasing a key; B: Flight Time (FT), time interval between releasing a key and pressing the next one; C: Press-Flight Rate (PFR), the ratio between the HT of a key and the FT to the next one; Speed (SP), the Euclidean distance X (in millimetres) between successive keys divided by FT.

The proposed method aims to test the discrimination potential of typing-related characteristics and yield a machine learning-based pipeline for classifying subjects as with or without DT, by leveraging data captured in-the-wild, during routine smartphone touchscreen typing. A remote study was conducted to capture the relevant data, during which participants installed a custom application (TypeOfMood) on their smartphone, completed an in-app digitised version of the PHQ-9 questionnaire, and used a custom software keyboard for typing, replacing their device default input method. By setting a cutpoint of PHQ-9 score equal to 5, representing mild levels of depressive symptoms^14^, study participants’ self-reported PHQ-9 compound score was used to categorize them in the two groups of interest, i.e., subjects with (PHQ-9 score ≥5) and without (PHQ-9 score < 5) DT. Typing-related characteristics included typing metadata (delete rate, number of characters typed and typing session duration) and keystroke dynamics, i.e., the detailed timing information of when keys are pressed and released. The focus was placed on two traditional variables of keystroke dynamics, i.e. the hold time (HT - time interval between pressing and releasing a key) and flight time (FT - time interval between releasing a key and pressing the next one), along with two novel variables, speed (SP - the distance between successive keys divided by the flight time) and press-flight rate (PFR - the ratio between the HT of a key and the FT to the next one). A representative illustration of the variables is provided in Fig. 6. Typing metadata, along with second- (median, standard deviation) and higher-order (kurtosis, skewness) statistics of the keystroke dynamics variables, extracted on a typing session level, are used to train/test, under a leave-one-subject-out (LOSO) cross-validation scheme, a feature selection and classification pipeline that eventually aggregates prediction probabilities extracted on session level to reach the final decision on a subject’s status, i.e., with or without DT.

### Study procedures

Data were collected via a remote study conducted with a mobile application, namely TypeOfMood. The study protocol was approved by the American Center of Psychiatry and Neurology of Abu Dhabi (Bioethics Committee, approval IRB reference 0022 ACPN). Electronic informed consent was obtained from all subjects prior to their participation in the study, via the application. Subjects held the right to withdraw from the procedure at any time, without providing any justification. Recruitment and study procedures were carried out according to institutional and international guidelines on research involving adult human beings.

### Study cohort

In order to recruit participants in the study, a series of intensive dissemination activities were undertaken by the Khalifa University of Science and Technology and the American Center for Psychiatry and Neurology, Abu Dhabi UAE, reaching out to the general population. From all participants, the ones finally included in the study were selected as being adults, less than 40 years old, matched in age and gender, without undergoing any medication treatment and without any upper limb dysfunction or other psycho-motor impairment. Moreover, eligible participants came from balanced catchment areas, had at least college education background, and were experienced in text messaging and smartphone use for more than one year. After installing the TypeOfMood application and providing consent, each participant answered a digitized version of the PHQ-9 questionnaire^14^ at first launch. From the total of 31 users that downloaded the application, six did not use the keyboard long enough to provide us with at least 50 keyboard sessions and therefore, they were omitted from the dataset, which eventually consisted of data provided by 25 participants.

The PHQ-9 questionnaire was chosen to be the evaluation criterion, as it is self-administrative, takes just a few minutes to fill and has sensitivity for major depression of 88%, specificity of 88%, for scores ≥10 and an overall sensitivity of 84%, specificity of 72% and 0.95 AUC^13,14^. Self-reported PHQ-9 scores were *a posteriori* used to categorise study participants into two classes, i.e., subjects with DT (n = 11) and HC (n = 14), based on the standardized PHQ-9 cutoff^42^ for mild or worse depressive symptoms, i.e., ≥5 and <5, respectively. Demographic and clinical characteristics of the study cohort are tabulated in Table 3.

**Table 3:** Study cohort characteristics with respect to the two groups.

Gray	Healthy Controls	Subjects with DT	Statistical Significance
*n* (total = 25)	14	11	N.A.
sessions (total = 37513)	20556	16957	n.s. (*p* = 0.98)
sessions per day (std)	66.46 (42.54)	55.14 (50.08)	n.s. (*p* = 0.49)
**Demographics**			
Women # (%)	6 (42.86%)	4 (36.36%)	n.s. (*p* = 0.77)
Men # (%)	8 (57.14%)	7 (63.64%)	n.s.(*p* = 0.77)
Avg. Age (std)	23.86 (4.44)	23.55 (3.24)	n.s. (*p* = 1.00)
Subjects who completed Education Level H/U #/#	10/4	9/2	n.s. (*p* = 0.55)
**Patient Health Questionnaire (PHQ-9) score**			
Avg. PHQ-9 (std)	2.29 (1.73)	10.64 (3.47)	**sig**. (*p* < 0.001)

The two groups formed by subjects with/without depressive tendency were fairly matched in terms of demographics, with no statistically significant differences (*p* < 0.05) observed, except for the PHQ-9 score, which was the criterion for the subject categorization. Statistical significance was computed using the two-sided Mann-Whitney U test. N.A.: not applicable; sig.: significant; n.s.: non-significant.

### Dataset acquisition

Participants installed the TypeOfMood application for Android OS, via Google’s Play Store, on their personal mobile phone. A Youtube video with instructions was available (https://youtu.be/-egEMpD12KE), in case they required help for the set-up of the application. The application was developed by the authors and included a custom software keyboard, similar to the Android OS default keyboard, including all modern functionalities, such as word prediction and auto-correction. Upon installation, participants were asked to provide demographic information and answer the PHQ-9 Questionnaire. After this initial setup, the default keyboard was replaced by the TypeOfMood keyboard and participants were able to use it in any application that evokes the keyboard. The software behind the keyboard was based on the Android Open Source Project keyboard, which was modified to capture keystroke-related data (key pixel coordinates and timestamps of key presses and releases), as well as typing metadata, i.e., number of deletes, number of characters typed, typing session duration, deliberate long-press events, and the application where the user typed, while the content of the typed text was not recorded at any point. Participants had the option to withdraw from the study at any point, without further explanations, either by uninstalling the application or by formally withdrawing their consent via the respective in-app option. All data captured by the application were temporarily stored locally on the mobile device and when Wi-Fi connection was available, they were transmitted to a secure Microsoft Azure Server for further processing. The data collection period lasted 124 days (from 2018-11-09 until 2019-03-13) and was concurrent with remote participant recruitment.

### Feature vector extraction

Let ${t}_{n}^{p}$ and ${t}_{n}^{r}$, *n* = 1, 2, …, *N*, be monotonically increasing time-stamp sequences corresponding to key press and release events, respectively, where *N* is the total number of keys pressed during a typing session. Let (*X*, *Y*) be the pixel coordinates of each key pressed, used for calculating vector *D* of Euclidean distances between consecutive keystrokes during a session. It must be noted that coordinates of keys were temporarily recorded on the mobile device and were not transmitted to the server. Instead, vector *D* was locally computed and transmitted, making it impossible for the researchers to know which key was pressed.

Time-stamp and distance sequences were used to derive the keystroke dynamics variables of interest, i.e., *HT*, *FT*, *SP* and *PFR*, for each typing session, along with their second-/higher-order statistics. Only sessions with at least eight characters typed were considered valid and all the rest were omitted from the subsequent analysis. The sequences of HTs and FTs are defined as $H{T}_{n}={t}_{n}^{r}-{t}_{n}^{p}$, n = 1*, 2, …, N* and $F{T}_{n}={t}_{n+1}^{p}-{t}_{n}^{r}$, n = *1, 2, …, N* − *1*, respectively. Distance sequences are defined as ${D}_{n}=\sqrt{{(\frac{({X}_{n+1}-{X}_{n})}{ScreenDensityX}\ast 25.4)}^{2}+{(\frac{({Y}_{n+1}-{Y}_{n})}{ScreenDensityY}\ast 25.4)}^{2}}$, n = *1, 2, …, N − 1*, where *ScreenDensity X* and *ScreenDensity Y* are the ratios of pixels per inch in the X and Y axis of the smartphone screen in portrait orientation, respectively, as obtained from the Android OS. SP and PFR sequences are defined as *SP*_*n*_ = *D*_*n*_/*FT*_*n*_ and *PFR*_*n*_ = *HT*_*n*_/*FT*_*n*_, n=*1, 2, …, N*, respectively.

In order to avoid data noise due to cases where the keyboard remained on-screen without any typing activity, e.g. user is waiting for message to answer back, resulting to high intervals between keys, FT sequences were conditionally filtered, with values larger than three seconds being removed. Additionally, FT values ≥0 were omitted from the sequences - negative values occur when the user presses a key before releasing the previous one, during two-handed typing. Furthermore, to minimize typing variability caused by external factors, such as walking and typing, we excluded outlier FT values where $|{\tilde{\mu }}_{n}^{FT}-\mu | > 3\ast \sigma$, where *μ* is the mean and *σ* is the standard deviation of FT median values of each individual user. HT values exceeding a threshold (usually 300 ms) were flagged as deliberately long-pressed keys and were excluded from the respective sequences.

Let *a*^*i*^ be any valid sequence of the *HT*, *FT*, *SP* and *PFR* sequences, after conditional filtering. Statistical features extracted to represent the *i*-th typing session in terms of keystroke dynamics are:

- *Median* ${\tilde{\mu }}_{i}=\frac{{a}_{\lceil \#n\div2\rceil }^{i}+{a}_{\lfloor \#n\div2+1\rfloor }^{i}}{2}$, where $\lceil x\rceil$ represents the least integer greater than or equal to *x* and $\lfloor x\rfloor$ the greatest integer less than or equal to *x*,
- *Standard deviation* $${\sigma }_{i}=\sqrt{\sum _{k}\,\frac{{a}_{k}^{i}-{\mu }_{i}}{N}},$$
- *Skewness* $${S}_{i}=\frac{{\sum }_{k}\,{(\frac{{a}_{k}^{i}-{\mu }_{i}}{{\sigma }_{i}})}^{3}}{N+1}$$ and
- *Kurtosis* $${K}_{i}=\frac{{\sum }_{k}\,{(\frac{{a}_{k}^{i}-{\mu }_{i}}{{\sigma }_{i}})}^{4}}{N+1}$$.

A feature vector *u*_*i*_ is then created as ${u}_{i}=\mathop{\cup }\limits_{a}\,[{\tilde{\mu }}_{i}^{a},{\sigma }_{i}^{a},{S}_{i}^{a},{K}_{i}^{a}]$ where *a* ∈ {*HT*, *FT*, *SP*, *PFR*}. Feature vector *u*_*i*_ and typing metadata, i.e., typing session duration (*T*_*i*_ - defined as the time elapsed between a launch and subsequent closing of the keyboard), total number of characters typed (*L*_*i*_), delete rate (*DR*_*i*_ - defined as the ratio of’Delete’ key presses over the total number of keys pressed), and characters typed per minute (*CPM*_*i*_), are combined to form a feature vector ${v}_{i}={u}_{i}{\cup }^{}[{T}_{i},{L}_{i},D{R}_{i},CP{M}_{i}]$ of size 20, representing the *i*-th session.

### Classification methodology

**Figure 7:**
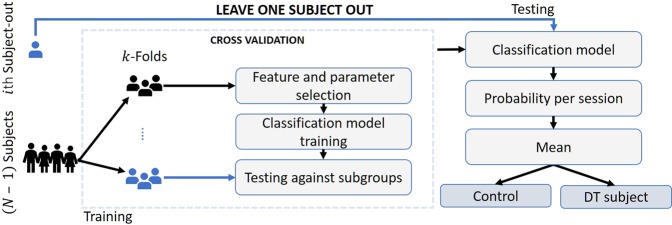
Illustration of the training and testing procedure of each leave-one-subject-out iteration. The *N* − 1 training subjects of each loop are used for feature selection and hyperparameter optimization, with a nested 5-fold cross-validation, and for training the classification model. Data of the *i*_*th*_ subject are used to test the trained classification model, with the mean of the outputted probabilities for all typing sessions yielding the decision on the subject’s status (subject with DT or healthy controls). The process is repeated until all subjects are left out and tested.

A leave-one-subject-out (LOSO) scheme was adopted for training and validating the classification pipeline developed (Fig. 7). In each iteration of the LOSO scheme, the *i*^*th*^ subject is left out and used as a test case, while data from the remaining *N* − 1 subjects are used to: (a) select the most discriminant features, (b) tune the classifier’s hyperparameters and (c) train the classification model. Regarding the training step of each LOSO iteration, feature vectors *v*_*i*_ of N − 1 subjects are initially fed into a nested 5-fold cross-validation to select the most discriminant features and optimize the classifier’s hyperparameters. In particular, training data are segmented into five folds, with four folds used for feature selection and grid search-based hyperparameter tuning of the classifier and the remaining fold to test the performance of resulting features and tuned classifier. Feature selection is performed by a select-*k*-best algorithm using the analysis of variance (ANOVA) *F*-value as the measure of feature importance, with an upper limit set to five (*k* = 5) regarding the number of features selected (to minimize the curse of dimensionality^57^). The process is repeated until all folds are used for testing. The selected features and classifier hyperparameters, which yield the highest AUC out of the five folds, form the classification model that is finally trained with the optimised feature vectors *v*_*i*_ of the N − 1 subjects. At the testing step of the LOSO iteration, data of the *i*^*th*^ left-out subject are fed to the trained feature selection and classification model, which outputs a probability score for each of her/his typing sessions. Finally, outputted probabilities are averaged and the mean probability is used to reach a final decision on the *i*^*th*^ subject’s status, i.e., subject with DT or HC. The LOSO iterative process stops when all subjects are used as test cases. Based on the aforementioned process, we tested the classification pipeline with three types of classifiers, i.e., Support Vector Machines^33^, Random Forest^34^ and Gradient Boosting^35^.

### Classification performance evaluation

ROC analysis was employed as the performance evaluation tool for the binary classification pipeline proposed. The analysis involves the iterative testing of specificity and sensitivity of a classifier against varying discrimination thresholds. To enhance the statistical robustness of the classification results, sampling with replacement (1,000 bootstraps) is further used here. The average value and 95% confidence interval of the area under the ROC curve (AUC) over 1,000 bootstraps are used as the key metrics to assess the performance of each classification setting. Where reported, sensitivity/specificity values are estimated by maximizing the Youden Index^58^, under the assumption of equal misclassification cost.

## Data Availability

All data generated and analysed during this work are available from the corresponding author on a reasonable request.
